# Long-Term Adherence to Levothyroxine Replacement Therapy in Thyroidectomized Patients

**DOI:** 10.3390/jcm11154296

**Published:** 2022-07-24

**Authors:** Raffaella Bocale, Giovambattista Desideri, Angelina Barini, Annamaria D’Amore, Mauro Boscherini, Stefano Necozione, Celestino Pio Lombardi

**Affiliations:** 1 Division of Endocrine Surgery, “Agostino Gemelli” School of Medicine, University Foundation Polyclinic, Catholic University of the Sacred Heart, 00198 Rome, Italy; raffaella.bocale@policlinicogemelli.it (R.B.); annamaria.damore@policlinicogemelli.it (A.D.); mauro.boscherini@policlinicogemelli.it (M.B.); celestinopio.lombardi@unicatt.it (C.P.L.); 2 Department of Life, Health and Environmental Sciences, University of L’Aquila, 67100 L’Aquila, Italy; stefano.necozione@univaq.it; 3 Institute of Biochemistry and Clinical Biochemistry, Department of Laboratory Medicine, “Agostino Gemelli” School of Medicine, University Foundation Polyclinic, Catholic University of the Sacred Heart, 00198 Rome, Italy; angelina.barini@policlinicogemelli.it

**Keywords:** adherence, levothyroxine, thyroid replacement therapy, thyroidectomy

## Abstract

(1) Background: We evaluated the long term adherence to two distinct formulations of levothyroxine (L-T4), liquid or solid, which are differently influenced by concomitant food ingestion. (2) Methods: A total of 106 thyroidectomized patients (82 female, mean age 58.2 ± 13.3 years) on L-T4 replacement therapy in either liquid (*n* = 52) or solid formulation (*n* = 54) were administered the four-item Medication Adherence Questionnaire (MAQ). (3) Results: The study population had 59.4% adherers and 40.6% non-adherers. The global MAQ score was significantly better in patients under liquid L-T4 in comparison to those under solid L-T4 (0.42 ± 0.82 vs. 0.83 ± 0.95, respectively, *p* = 0.0085). The patients on tablet L-T4 forgot to take their medication more frequently than those on liquid LT4 treatment (*p* = 0.0159) and were more often careless at times about taking their medication (*p* = 0.007), whilst about one in two thyroidectomized patients preferred tablets for lifetime medication therapy. The global MAQ score was directly correlated with the circulating TSH levels in the whole study population (0.700, *p* < 0.0001) and inversely correlated with both the FT3 (−0.220, *p* = 0.0232) and FT4 (−0.327, *p* = 0.0006) serum concentrations. (4) Conclusions: Long-term adherence to L-T4 treatment was globally satisfactory although it was better for the liquid formulation, which appears to represent an easier-to-manage L-T4 replacement therapy for most thyroidectomized patients, particularly for those with difficulties in taking L-T4 while fasting.

## 1. Introduction

Following thyroid surgery, thyroid hormone replacement therapy is used to replace deficient thyroid hormones and to prevent postoperative thyroid hypofunction [1,2,3]. The goal of post-thyroidectomy replacement therapy is to restore thyroid function, avoiding over- and undersubstitution by beginning with an ideal dose of levothyroxine (L-T4) within seven days after surgery [4]. 

By definition, an optimal thyroid hormone replacement should be able to restore thyroid stimulating hormone (TSH) concentrations to a normal value, with normal or slightly increased serum thyroxine concentrations. Some patients experience difficulties in achieving an adequate thyroid hormone profile under replacement therapy [1]. Poor medication adherence is the main cause of low efficacy of pharmacological therapy, and it is more common in chronic diseases. 

For example, among hypothyroid patients, it was estimated that, after 5 years of levothyroxine therapy 21.5% of patients still have a TSH level > 5.0 mU/L due to poor medication adherence [5]. In addition to the traditional poor adherence represented by skipping one or more doses, the low adherence to the modality of assumption of levothyroxine could influence L-T4 bioavailability, thus, impairing the efficacy of replacement therapy. In this regard, the classical formulation of L-T4 is a tablet containing a sodium salt of L-T4 together with different excipients [6]. A number of exogenous conditions affect L-T4 intestinal absorption, including beverages [7,8]. 

According to these pharmacokinetic features, the maximum absorption of L-T4 occurs after the overnight fasting in the morning. The lowest absorption occurs when the drug is taken together with breakfast [9,10]. Recent evidence has demonstrated a more favorable pharmacokinetic profile of the liquid and soft gel formulations of L-T4 in comparison to traditional tablets, allowing for better L-T4 absorption [11,12]. Indeed, these new L-T4 formulations bypass the pH-depending dissolution phase and are less dependent on the binding with sequestrants in the intestinal lumen [11]. 

In this regard, we recently described a greater efficacy of liquid formulation L-T4 in ameliorating the thyroid hormone profile after 2 months under replacement therapy in recently thyroidectomized patients. This improvement was paralleled by an amelioration of mood states and self-perception of mental well-being [13]. These findings could suggest that the favorable efficacy profile of liquid formulation of L-T4 could encounter a greater appreciation by patients, thus, potentially leading to higher adherence. 

In this regard, a valuable cross-sectional study by Cappelli et al. [14] recently demonstrated a high adherence to L-T4 treatment for both tablet and liquid formulations. Taking L-T4 at breakfast was considered the most convenient option for most patients [14]. Starting from these evidence, in the current report, we evaluated the long term adherence to either liquid or tablet L-T4 replacement therapy among thyroidectomized patients previously enrolled in a randomized prospective intervention trial by our group [13] about seven years after the conclusion of the study and the return of patients to usual care management. 

## 2. Materials and Methods

### 2.1. Patients

One hundred six thyroidectomized patients (82 female and mean age 58.2 ± 13.3 years), were analyzed in the current research. All subjects participated about 7 years ago to a 2 months intervention study from our group aiming to compare the different influence of L-T4 replacement therapy in either liquid or tablet formulations on mood states, self-perceived psychological well-being and thyroid hormone profile in a cohort of recently thyroidectomized patients [13]. 

Briefly, these patients were initially randomized to fifth-to-seventh day after total thyroidectomy to receive an individualized dose of L-T4 in liquid or tablet formulation for 2 months with the recommendation to take the drug early in the morning, at least 60 min before consumption of any solid and/or liquid food. After the conclusion of this previous intervention study, all patients were invited to continue the replacement therapy regimens followed during the trial and to return to usual care management. About seven years after the study conclusion, 106 subjects were still followed in our Outpatient Unit and were evaluated in the current study with regard to medical adherence and thyroid hormone profile.

### 2.2. The Four-Item Medication Adherence Questionnaire (MAQ)

The study participant’s medication adherence was assessed by the MAQ, a self-report questionnaire with four questions/items. The respondents were asked to answer yes or no regarding items that assess the participants’ history of medication adherence [15]. Items were scored as either 0 (no) or 1 (yes), and previous investigations have typically summed all items to report a total score. The four-item MAQ has been well-validated to identify adherence behavior in a number of chronic conditions, and scores have been shown to correlate well with objective adherence measures and clinical outcomes. 

Indeed, individuals who scored in the high adherence range had a significantly better treatment outcomes than those scoring in the low adherence range as measured by the MAQ [15]. Participants answering “no” to all items of the MAQ (score = 0) were identified as adherers to their medicine, while participants answering “yes” to at least one of the MAQ items (score = 1–4) were identified as “non-adherers”. This cut-off has been used in the literature and provides a highly sensitive tool for identifying medication non-adherence.

Two further questions specifically regarding the two L-T4 formulations considered in the current report were also administered at the end of the four standard items to specifically evaluate preferences between the tablet and liquid forms of L-T4 for lifetime treatment [14]: (1) “Do you prefer to take drugs in liquid or solid formulation” with three answer options “liquid”, “solid” and “indifferent”; (2) “Is it a problem for you to take your medication while fasting or at least 30 min before having breakfast or coffee?” with two answer options “yes” or “no”.

### 2.3. Laboratory Analysis

Blood samples were drawn from each participant after an overnight fasting period for determination of the circulating levels of thyroid hormones. TSH, free T3 (FT3) and free T4 (FT4) were measured by COBAS 600 (Electrochemiluminescence Technology, Roche Diagnostics, Mannheim, Germany).

### 2.4. Statistical Analysis

SAS version 9.4 was used to perform the statistical analysis. The normality of the distributions of variables were evaluated with the Shapiro–Wilk test. Comparisons between variables were performed with Wilcoxon Rank Sum test due to the non-normal distribution of data. The Chi squared test or Fisher’s Exact Test, as appropriate, were used to compare categorical variables. Spearman non-parametric correlation was used to evaluate correlations between variables. If it is not otherwise specified, data are presented as the mean ± SD. A cross-tabulation was done based on the MAQ to differentiate the respondents as adherent (answered ‘‘no’’ to all items of the MAQ scale), intentionally non-adherent (answered ‘‘yes’’ to stopped taking medications when felt better or worse about health), unintentionally non-adherent (answered ‘‘yes’’ to forgetful or careless about taking medications) and mixed group (answered ‘‘yes’’ for both intentional and unintentional adherence items) [16]. 

## 3. Results

Among the 106 subjects that are included in the current report, 54 were originally assigned to the liquid formulation of levothyroxine and 52 to the solid formulation. During the follow-up period (87.7 ± 3.3 months), three participants shifted from the liquid to solid formulation, and one had shifted from the solid to liquid formulation. Thus, the current analysis was performed by comparing the study outcomes between a group of 52 subjects (40 females and 12 males, mean age 58.6 ± 12.7 years) taking the liquid formulation of L-T4 and 54 subjects (42 females and 12 males, mean age 57.9 ± 13.9 years) taking the solid formulation of L-T4. The slight but significant difference in the thyroid hormone profiles between treatment groups under liquid or tablet L-T4 replacement therapy that we described at the end of the previous intervention study [13] was no longer evident in the cohort of subjects included in the current report likely because of the smaller sample size (Table 1). On the other hand, in the current cross-sectional analysis, patients under liquid L-T4 replacement therapy had significantly lower circulating levels of TSH and significantly higher serum levels of FT4. 

The responses to the MAQ are reported in Table 2. Using the already defined cut-off, this study population had 59.4% adherers (48.2% on tablets and 71.2% on liquid) and 40.6% non-adherers (51.8% on tablets and 28.8%% on liquid, respectively). Among non-adherers, the large majority of subjects scored 1 (55.8%) or 2 (37.2%) on the adherence scale, indicating a low grade of non-adherence. According to this, the global MAQ score was quite low (0.63 ± 0.91) in the whole study population with a better adherence score in patients under liquid L-T4 in comparison to those under tablet L-T4 (0.42 ± 0.82 vs. 0.83 ± 0.95, respectively, *p* = 0.0085). 

The patients on tablet L-T4 forgot to take their medication more frequently than those on liquid LT4 treatment (51.9% vs. 28.9% *p* = 0.0159) and were more often careless at times about taking their medication (27.8% vs. 7.7% = 0.007). The cross-tabulation of the MAQ items (Table 3) demonstrated that, among the non-adherers, about 93% considered themselves as unintentional non-adherers and 7% as mixed non-adherers, while none of them were identifiable as intentional non-adherers.

Due to the early shift of a few patients from one to the other L-T4 formulation, and vice versa, we performed an additional analysis by considering the original assignment to the two forms of L-T4 replacement therapy (54 subjects under liquid and 52 under solid L-T4). The TSH and FT4 values were still better in patients treated with liquid L-T4 in comparison to those on table L-T4 (*p* = 0.0089 and *p* < 0.0001, respectively). 

The MAQ score was still better in patients under liquid L-T4 in comparison to those under solid L-T4 (0.37 ± 0.73 vs. 0.90 ± 0.99, respectively, *p* = 0.0018), mainly because of a better pattern of answers to items 1 (*p* = 0.0063) and 2 (*p* = 0.0007). 

**Figure 1:**
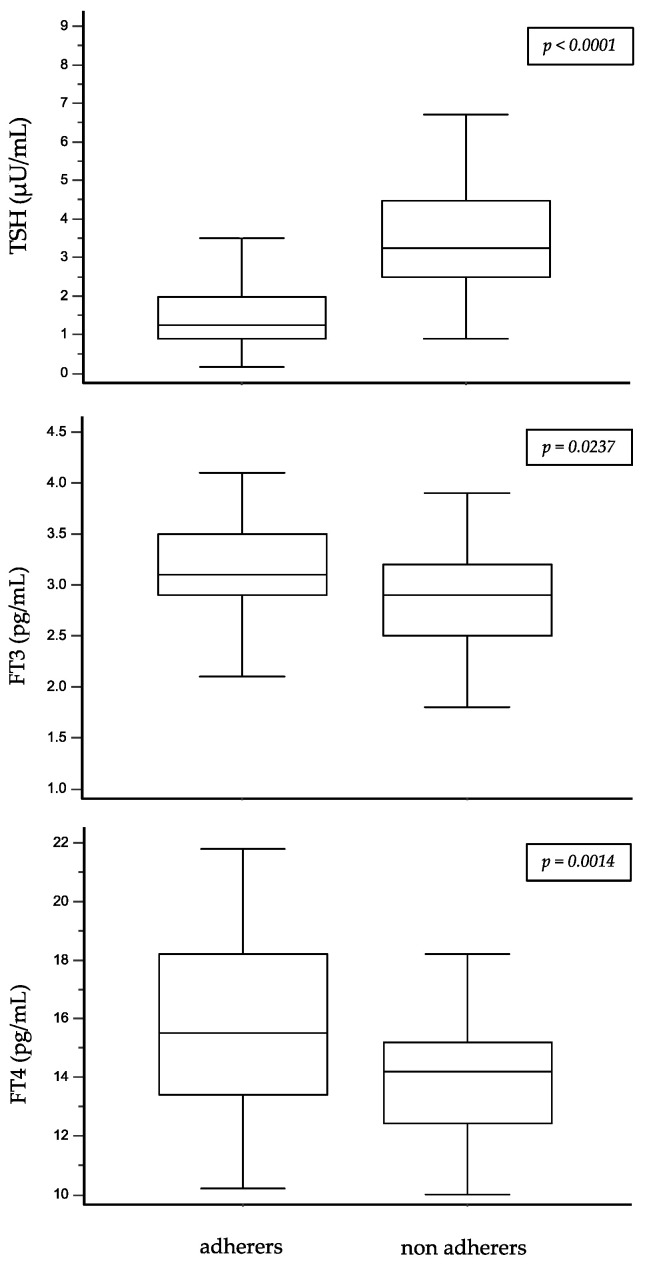
Circulating levels of TSH, FT3 and FT4 in adherers and non-adherers to L-T4 replacement therapy (Wilcoxon Rank Sum Test).

Considering the study population as a whole, adherers had lower circulating levels of TSH and higher circulating levels of FT3 and FT4 (Figure 1). The global MAQ score was directly correlated with the circulating TSH levels in the whole study population (0.700, *p* < 0.0001) and inversely correlated with both FT3 (−0.2220, *p* = 0.0232) and FT4 (−0.327, *p* = 0.0006) serum concentrations.

The two items administered to specifically evaluate medication preference between tablet and liquid LT4 formulations revealed that 56 of 106 patients (52.8%) preferred tablets for lifetime medication therapy even though taking the tablet at least 30 min before breakfast was a problem for 69 of 106 patients (65.1%). One among five participants (21 subjects, 19.8%) had no preference for either the solid or liquid L-T4 formulations.

## 4. Discussion

The result of this study clearly demonstrated satisfactory long-term adherence to L-T4 replacement therapy in thyroidectomized patients with 59% of patients who were identifiable as fully adherers (MAQ score = 0) and 38% as medium adherers (MAQ score = 1–2) while only 3% of subjects exhibited poor adherence (MAQ score = 3–4). Interestingly, some significant differences in term of adherence between tablet and solid L-T4 formulations were observed. Indeed, the global MAQ score was worse in patients on tablet L-T4 mainly because they forgot to take their medications more frequently than those on liquid L-T4 treatment and were more often careless at times about taking their thyroid medication. 

L-T4 treatment is one of the most frequent therapies worldwide because of the great diffusion of thyroid disorders requiring L-T4 replacement therapy and its relative simplicity to adjust. Many surveys on hypothyroidism showed that about half of patients are either under- or over-treated [17,18]. Impairment of the intestinal absorption of L-T4 due to food, drugs and diseases could contribute to explaining this incomplete therapeutic success [19]. However, poor adherence to medications should be also considered. Indeed, low adherence with self-administered medication is widespread [20,21]. This affects all chronic conditions with adherence rates averaging at 50% [20,21]. Non-adherence with medication undermines the benefits of medical therapy depending on both the efficacy of treatment and the actual level of non-adherence, and it also affects health care costs. During the last years, few studies evaluated therapeutic adherence to L-T4 replacement therapy in different clinical contexts and by using different methodological approaches. Briesacher et al. [22], using health care claims data, demonstrated a high level of adherence among patients with hypothyroidism with a medication possession ratio ≥ 80% in 68.4% of subjects. A recent report by Vezzani et al. [23] reported medium-high adherence in patients with newly diagnosed primary hypothyroidism with 23.9% low, 38.6% medium and 37.5% highly adherers. Similar results were found by Juch et al. [24] among pregnant women with hypothyroidism; adherence was low among 17% of medicated women, whilst it was moderate and high among 44% and 39% of medicated women, respectively. 

In the current report, we provide clear evidence of satisfactory medication adherence to L-T4 replacement therapy in a cohort of thyroidectomized patients as indicated by the high percentage of subjects who were full adherers or medium adherers. These results were not unexpected because the cohort of subjects evaluated in the current report was derived from patients enrolled about 7 years ago in the previously cited prospective intervention study by our group [13]. Indeed, during the intervention study, these patients repeatedly received detailed information about the importance of good therapeutic adherence. In addition, since all the participants had hypothyroidism following total thyroidectomy, it is conceivable that these subjects might have developed a particular sensibility towards the optimal management of their clinical condition. According to this data interpretation, almost all non-adherers considered themselves as unintentional non-adherers [16]. This kind of patient typically has stronger beliefs that their health can be improved by healthcare providers [25]. 

The second interesting finding of our study was the demonstration of a significantly better MAQ score in patients under liquid L-T4 in comparison to those under tablet L-T4 although about one among two thyroidectomized patients (52.8%) preferred tablets for lifetime medication therapy. The patients on tablet L-T4 forgot to take their medication more frequently than those on liquid LT4 treatment and were more often careless at times about taking their medication. This relevant topic has only been previously addressed by few studies with somewhat conflicting results. In a large retrospective study, Hepp et al. [26] demonstrated that soft-gel L-T4 formulation was associated with significantly higher adherence compared to all other levothyroxine formulations at both 6 and 12 months. On the other hand, a recently published Italian survey regarding the diagnosis and clinical management of primary hypothyroidism did not find any difference in the adherence score among L-T4 formulations in relation to adherence categories [23]. Finally, the already cited study by Cappelli et al. [14] demonstrated a particularly high adherence to L-T4 replacement therapy (87.2%) in a cohort of 320 ambulatory care patients affected by hypothyroidism with no difference between the tablet and liquid formulations. However, despite the similar adherence to both L-T4 formulations, the percentage of subjects who sometimes forgot to take their medication was significantly higher in patients taking L-T4 tablets (64% vs. 34%, *p* < 0.0001) [13], as we found in our study. In addition, also the score related to the difficulty in remembering to take all their medication was significantly worse in patients taking L-T4 tablets [13]. It is interesting to observe that, in both studies, the majority of patients had a problem taking the tablet at least 30 min before breakfast, although more than half of the participants preferred the tablet L-T4 formulation for lifetime treatment.

The last interesting finding of our study was the association between the adherence score and thyroid hormone profile. Indeed, adherers had lower circulating levels of TSH and higher circulating levels of FT3 and FT4 in comparison to non-adherers. In addition, the global MAQ score was directly correlated with circulating TSH levels in the whole study population and inversely correlated with both the FT3 and FT4 serum concentrations. These results support the robustness of our data regarding the good therapeutic adherence of our study population and indicate good reliability of MAQ in assessing therapeutic adherence to thyroid hormone replacement therapy in thyroidectomized patients.

Taken together, our findings provide the interesting suggestion of a potential advantage deriving from liquid L-T4 replacement therapy in terms of adherence during a prolonged observation, which could be somewhat related to the more favorable pharmacokinetic profile of this formulation that allows the possibility of taking the drug with breakfast, leading to a significant improvement in daily pharmacologic treatment. The evidence towards a better thyroid hormone profile in patients under liquid L-T4 replacement therapy appears to confirm this data interpretation. In this regard, during the intervention phase of the study from which this cohort of patients was derived, participants were directed to take the drug early in the morning, at least 60 min before the con-sumption of any solid and/or liquid food, including coffee, whilst the adherence was frequently checked [13]. After the conclusion of the study, patients went back to routine follow-up, and the recommendation to take L-T4 preferably while fasting was no longer so frequently reinforced. It is interesting to observe that changing from L-T4 tablets to the liquid formulation at breakfast was associated with an improvement of the quality of life of the patients resulting in the perception of a greater convenience of the treatment [27]. In addition, we previously demonstrated the greater efficacy of the liquid formulation of L-T4 in ameliorating mood states and the self-perception of mental well-being, in addition to affecting the thyroid hormone profile, in recently thyroidectomized patients [13].

The results of the current report, although convincing, should be considered in the light of some possible study limitations. First, we analyzed data from patients who spontaneously decided to continue to be followed by our Outpatients Unit after the conclusion of our previous intervention study [13]. Thus, we cannot completely exclude that these patients could have been particularly motivated to be adherent to the prescribed therapy. In addition, we considered only one single thyroid function measurement. Thus, we cannot completely exclude the possibility that the fluctuations of thyroid function over time could have at least, in part, influenced our findings. However, the MAQ was administered close to the thyroid function assessment. This tight temporal relationship allows us to speculate that the relationship between the adherence score and the thyroid hormone profile could be the expression of the good reliability of MAQ in assessing the adherence to L-T4 replacement therapy. Finally, although significant statistical differences for TSH and FT4 were seen between the formulations, these differences were likely not clinically relevant, as all the values were in the normal ranges. However, these differences could be somewhat relevant as a confirmation of a different trend of adherence between the two formulations as suggested by the MAQ score results. 

## 5. Conclusions

Our data demonstrated that long-term adherence to L-T4 treatment was globally satisfactory although it was better for the liquid formulation, which appears to represent an easier-to-manage L-T4 replacement therapy for most thyroidectomized patients, particularly for those with difficulties in taking L-T4 while fasting. Furthermore, our data suggest that MAQ might represent an adequate tool for monitoring adherence to L-T4 replacement therapy in a real world setting.

## Figures and Tables

**Table 1:** Thyroid hormone profile of the study population.

	Baseline			Follow-Up		
	Liquid (*n*:52)	Tablet (*n*:54)	*p* *	Liquid (*n*:52)	Tablet (*n*:54)	*p* *
TSH (μU/mL)	2.1 ± 2.5	2.5 ± 2.3	0.168	2.0 ± 1.6	2.7 ± 1.6	0.007
FT3 (pg/mL)	3.0 ± 0.6	2.9 ± 0.7	0.336	3.1 ± 0.6	3.0 ± 0.6	0.200
FT4 (pg/mL)	15.5 ± 3.2	14.7 ± 2.9	0.392	15.9 ± 3.0	14.2 ± 2.3	0.002

* Wilcoxon Rank Sum test.

**Table 2:** The four-item MAQ.

	Tablet (*n*:54)	Liquid (*n*:52)	*p* *
	No/Yes	No/Yes	
1. Do you ever forget to take your medicine?	26/28	37/15	0.0159 *
2. Are you careless at times about taking your medicine?	39/15	48/4	0.007 *
3. When you feel better do you sometimes stop taking your medicine?	53/1	50/2	n.s. **
4. Sometimes if you feel worse when you take the medicine, do you stop taking it?	53/1	51/1	n.s. **

* Chi-squared Test; ** Fisher’s Exact Test.

**Table 3:** Cross-tabulation analysis of the MAQ.

		Intentional Non-Adherers ^a^	
		Yes	No
**Unintentional** **Non-Adherers ^b^**	yes	mixed (3)	unintentional (40)
	no	intentional (0)	adherers (63)

^a^ Stop taking medications either when feeling better or worse. ^b^ Stop taking medications due to forgetfulness or carelessness.

## Data Availability

The data are contained within the article.
